# An evaluation of tuberculosis contact investigations against national standards

**DOI:** 10.1136/thoraxjnl-2016-209677

**Published:** 2017-04-07

**Authors:** Sean M Cavany, Tom Sumner, Emilia Vynnycky, Clare Flach, Richard G White, H Lucy Thomas, Helen Maguire, Charlotte Anderson

**Affiliations:** 1 TB Modelling Group, TB Centre and CMMID, Faculty of Epidemiology and Population Health, London School of Hygiene and Tropical Medicine, London, UK; 2 Statistics, Modelling and Economics Department, Public Health England, London, UK; 3 Faculty of Epidemiology and Population Health, London School of Hygiene and Tropical Medicine, London , UK; 4 Respiratory Diseases Department, Public Health England, London, UK; 5 Field Epidemiology Services, Public Health England, London, UK; 6 Department of Infection and Population Health, University College London, London, UK

**Keywords:** Tuberculosis

## Abstract

**Background:**

Contact tracing is a key element in England's 2015 collaborative TB strategy, although proposed indicators of successful contact tracing remain undescribed.

**Methods:**

We conducted descriptive and multivariable analyses of contact tracing of TB cases in London between 1 July 2012 and 31 December 2015 using cohort review data from London's TB Register, identifying characteristics associated with improved indicators and yield.

**Results:**

Of the pulmonary TB cases notified, 60% (2716/4561) had sufficient information for inclusion. Of these, 91% (2481/2716) had at least 1 contact (median: 4/case (IQR: 2–6)) identified, with 86% (10 251/11 981) of these contacts evaluated. 4.1% (177/4328), 1.3% (45/3421) and 0.70% (51/7264) of evaluated contacts of pulmonary smear-positive, pulmonary smear-negative and non-pulmonary cases, respectively, had active disease. Cases who were former prisoners or male were less likely to have at least one contact identified than those never imprisoned or female, respectively. Cases diagnosed at clinics with more directly observed therapy or social workers were more likely to have one or more contacts identified. Contacts screened at a different clinic to their index case or of male index cases were less likely to be evaluated than those screened at the same clinic or of women, respectively; yield of active disease was similar by sex. 10% (490/4850) of evaluated child contacts had latent TB infection.

**Conclusions:**

These are the first London-wide estimates of TB contact tracing indicators which are important for monitoring the strategy's success and informing risk assessment of index cases. Understanding why differences in indicators occur between groups could improve contact tracing outcomes.

**Key messages**

**What is the key question?**

- In London, what is the baseline level of contact tracing indicators for the Public Health England and National Health Service England collaborative TB strategy?

**What is the bottom line?**

- In London 91% of pulmonary index cases have at least one contact identified and 86% of those identified are evaluated for signs of TB and latent infection; there were significant differences in these indicators between cases, including when grouped by the sex of the case, whether they have social risk factors, and the staffing levels of the clinic.

**Why read on?**

- These results provide an important baseline for monitoring progress of England's national TB strategy and highlight areas in which improvements can be made, particularly those which show improved indicators for contacts screened at the same clinic as their index case or clinics with a greater number of directly observed therapy (DOT) or social workers.

## Introduction

In 2015, there were 10.4 million incident TB cases and 1.4 million deaths worldwide, making TB the largest cause of death by a single infectious agent.^1^ While England is a low burden country, with 5758 notified cases in 2015, 39% of these occurred in London,^2–4^ where overall incidence was 26/100 000/year; in 2014, it reached 79/100 000/year in one London borough.^4^ While incidence remains highest among the foreign-born, and many cases are likely due to reactivation of infection acquired abroad, molecular epidemiological analyses attribute up to a third of new cases in London to recent transmission.^5^ UK-born patients with TB and those of white ethnicity, 25% of whom have social risk factors, were frequently ‘clustered’ and also more likely to have delays exceeding 4 months from symptom onset to starting treatment.^4^ The *Collaborative Tuberculosis Strategy for England*,^3^ published in 2015, highlights contact tracing as an important tool for improving early TB diagnosis and reducing transmission. Contact tracing, which seeks to identify and diagnose contacts of infectious cases, has been used for decades in high-income countries where the heightened risk of disease among contacts relative to the general population makes it effective.^6–8^ The strategy proposes two indicators of improved contact tracing: the proportion of pulmonary TB cases with close contacts identified, and the proportion of identified close contacts of pulmonary TB cases that are evaluated.

TB contact tracing in England broadly follows the stone-in-the-pond principle.^9^ Clinics evaluate close contacts (those with contact similar to that in a household) first, followed by casual contacts (eg, workplace or school contacts) if first round investigations suggest transmission to close contacts has occurred. In England, until 2016, guidance recommended identification and evaluation of household contacts of all index cases, after which tracing just household contacts of index cases with pulmonary or laryngeal disease was recommended.^7^ In 2010, cohort review, an approach to case management and contact investigation appraisal that was shown to improve case management outcomes, was first introduced to London, and occurs quarterly.^10^ ^11^

Several studies have found differences in both the proportion of TB contacts evaluated and the yield (the proportion of evaluated contacts diagnosed with TB) between ethnic groups and different disease types in the UK^12–14^ and elsewhere.^15–17^ The findings and understanding gained from such studies are not readily transferable to the large and ethnically diverse metropolis of London. Using data on contact tracing collected in London through cohort review since mid-2012, the main aim of this study is the presentation of baseline levels of the strategy contact tracing indicators, and of the proportion of contacts who are secondary cases or have latent TB infection (LTBI) (the ‘yield’). A secondary aim is the identification of demographic and clinical characteristics associated with different indicators and yield estimates.

## Methods

### Definition of terms

During the study period (1 July 2012–31 December 2015, see below), immediately after the diagnosis of the index case, the nurse asked the case for a list of close contacts. These contacts were then requested to attend for screening as soon as possible. Screening begins with symptom-screen; for asymptomatic contacts this is followed by a tuberculin skin test (TST) or interferon γ release assay (IGRA) in those under 35 years and consideration of a chest X-ray (CXR) in those 35 years and over.^7^ Those with a positive symptom-screening, TST/IGRA result or CXR are evaluated for signs of active TB. LTBI is defined as a positive result on either a TST or IGRA, which both have variable sensitivity,^6^ and the absence of active disease. Those with LTBI are considered for preventive therapy and/or BCG vaccination. The numbers of contacts with TB is recorded in the London TB Register (LTBR) at the initial contact investigation, so all new cases of TB among contacts can be considered prevalent.^18^ ^19^

### Data set and inclusion criteria

The primary data source was the LTBR, a web-based register containing demographic and clinical data on all TB cases notified in London since 2002. Clinical and demographic information on patients is entered directly to the LTBR by TB clinic staff. We restricted analyses to data collected after the introduction of cohort review (1 July 2012), after which the database includes data on the aggregate number of contacts per case that were: identified, evaluated, found to have LTBI and/or active TB. Contact data were aggregated by whether the contact was a child or adult (15 years old or above) and whether they were evaluated at the same clinic as the index case or elsewhere. As only contacts aged less than 35 years were evaluated for LTBI, data on LTBI status of contacts was only used for child contacts. No other demographic data on contacts were collected. Individual level data were not available for whether a home visit was undertaken; instead, the clinic's policy was used to provide clinic-level data. Only data from household and other close contacts are included in the analysis.

London is divided into five sectors; in some of these, a non-random selection of cases was reported at cohort review and recorded in the LTBR. To avoid bias, we only included cases in the analysis if the sector in which they were notified reported 80% or more of their cases at cohort review in a given quarter (defined as at least one of the cohort review fields completed) (see online supplementary appendix for a description of included quarters by sector). Selection of quarters was done separately for analyses including all cases, or just pulmonary cases (see online supplementary appendix). We removed further cases if: their line listing contained inconsistencies (eg, more contacts evaluated than were identified); included contacts were probably casual contacts (an incident was declared and more than 20 close contacts were identified); a patient was not reported to cohort review; or the index was detected through a previous contact investigation. In multivariable analyses, we also removed cases if data on an included exposure were missing. If the field describing number of contacts was missing, we assumed it was zero if other cohort review fields were complete and this assumption did not create inconsistencies.

10.1136/thoraxjnl-2016-209677.supp1 Supplementary data

### Indicators

We calculated the following four indicators:

1. The proportion of pulmonary index cases who had at least one contact identified;
2. The proportion of identified contacts of pulmonary index cases who were evaluated;
3. The proportion of all evaluated contacts that had active TB (‘Yield of active disease per contact’);
4. The proportion of all evaluated child contacts that had LTBI (‘Yield of LTBI per child contact’).

Indicators 1 and 2 were proposed in the National Health Service England and Public Health England collaborative strategy^3^ and are recommended in the US Centers for Disease Control and Prevention guidelines,^20^ while indicators 2, 3 and 4 were used in a systematic review of European contact investigations.^15^ As WHO and European Centre for Disease Prevention and Contro guidelines refer to low-income/middle-income countries and multidrug-resistant cases, respectively, we did not focus on these indicators here.^21^ ^22^ Indicators 1 and 2 include only pulmonary index cases, whereas indicators 3 and 4 include all index cases, in order to estimate the yield from screening contacts of non-pulmonary cases. While LTBI treatment is an important outcome of contact tracing in England, it is not covered in this article.

### Statistical analysis

We calculated the four indicators London-wide and for subsets of index cases (based on disease site, smear status, age of contact and ethnic group). We used multivariable logistic regression to assess the association of clinical and demographic factors of the index cases, contacts, clinics and local authorities with whether or not the index case (indicator 1) or contact (indicators 2–4) satisfies each of the four indicators (see online supplementary table S1 for details of included variables). As this was an exploratory study all clinical and demographic factors with a plausible direct or confounding impact on the outcome were included in the model (see online supplementary appendix for the exposures included). We assessed all variables for multicollinearity. For indicators 2–4, as each index case may have several contacts, index case exposures tend to cluster; adjustments were made to the p values and CIs to account for this, using the between-cluster variance estimator in Stata.^23^ For indicator 4, we excluded adult contacts, as we did not know which of them were tested for LTBI.

Note that there may be discrepancies between numbers presented for the levels of the indicators and for the multivariable analysis, due to cases missing data on variables included in the multivariable analysis. All data were analysed using Microsoft Excel V.14.0 and Stata V.13.1.

## Results

### Comparison of included and excluded cases

**Figure 1:**
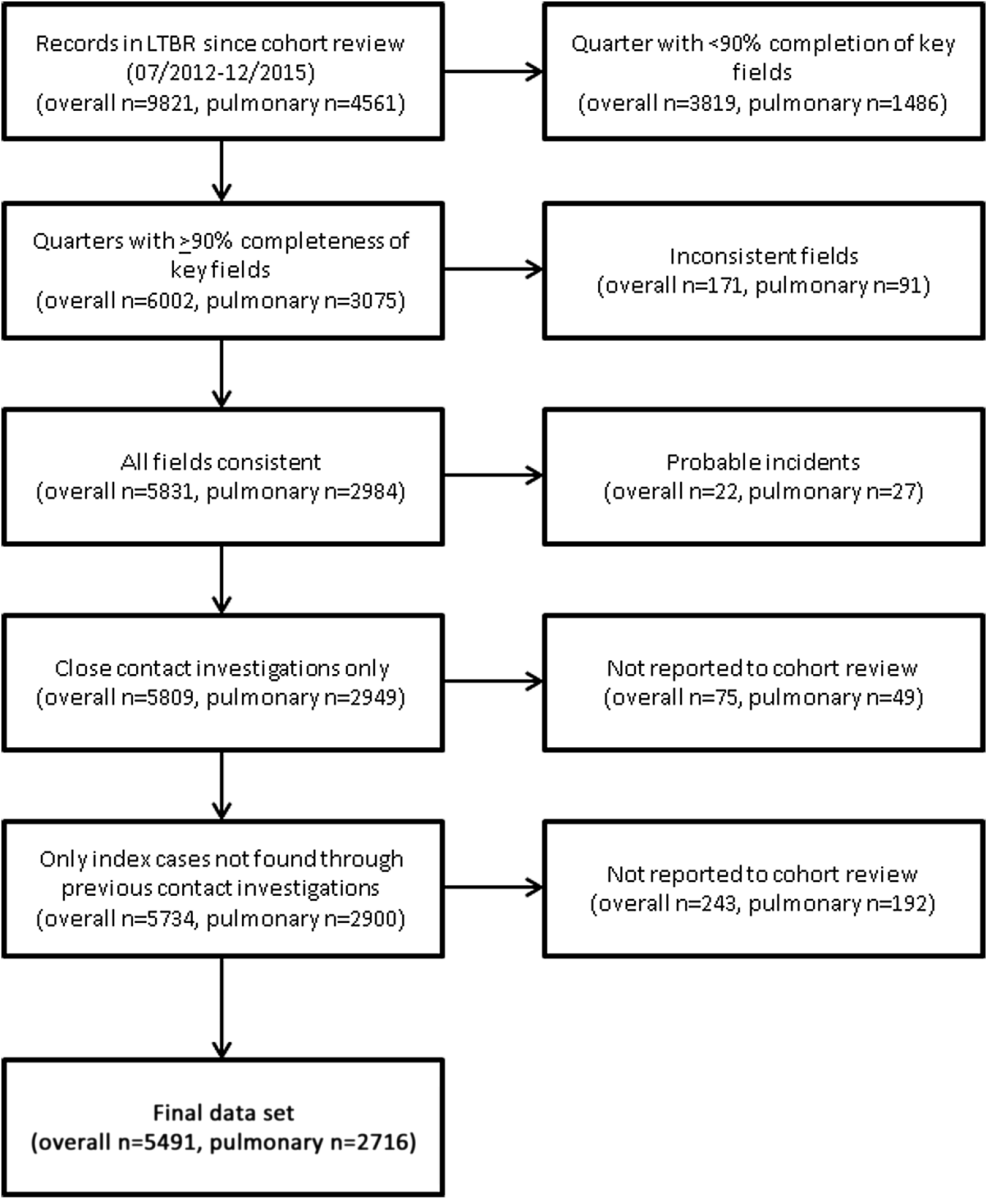
Description of included and excluded cases. LTBR, London TB Register.

From 1 July 2012 to 31 December 2015 inclusive, 9821 cases were reported in the LTBR of which 4561 were pulmonary. After excluding cases, 5491 cases of all forms of TB and 2716 pulmonary cases remained (figure 1). When considering all cases, there were 971 (18%) pulmonary smear-positive cases, 1095 (20%) pulmonary smear-negative cases, 478 (8.7%) pulmonary cases with unknown smear, four laryngeal cases without pulmonary involvement and 2943 (54%) non-pulmonary, non-laryngeal cases. In general, included and excluded cases shared similar clinical and demographic factors (table 1).

**Table 1:** Comparison of characteristics of cases that were included and excluded in the analyses. Percentages are within-group column percentages with the exception of the total row, where row percentages are given. Within-group totals may be discrepant to the stated total due to cases with missing data

Factor	Index cases with all forms of TB		Index cases with pulmonary TB	
	Number included (%)	Number excluded (%)	Number included (%)	Number excluded (%)
Total	5491 (56%)	4330 (44%)	2716 (60%)	1845 (40%)
UK-born?				
Yes	830 (19%)	943 (17%)	622 (23%)	500 (27%)
No	2880 (67%)	3862 (70%)	1744 (64%)	1093 (60%)
No, recent migrant (<2 years)	578 (13%)	674 (12%)	344 (13%)	233 (13%)
Ethnicity				
Bangladeshi	257 (5%)	317 (7%)	105 (4%)	85 (5%)
Black-African	1278 (23%)	726 (17%)	608 (22%)	361 (20%)
Black-Caribbean	208 (4%)	118 (3%)	116 (4%)	66 (4%)
Black-Other	82 (2%)	58 (1%)	40 (2%)	29 (2%)
Chinese	81 (1%)	37 (1%)	52 (2%)	19 (1%)
Indian	1330 (24%)	1434 (33%)	510 (19%)	461 (25%)
Pakistani	976 (18%)	607 (14%)	496 (18%)	290 (16%)
White	498 (9%)	525 (12%)	201 (7%)	189 (10%)
Other	758 (14%)	476 (11%)	578 (21%)	330 (18%)
Sex				
Male	3287 (60%)	2537 (59%)	1704 (63%)	1122 (61%)
Female	2204 (40%)	1793 (41%)	1012 (37%)	723 (39%)
Site of disease				
Pulmonary or laryngeal	2548 (46%)	2018 (47%)	N/a	N/a
Non-pulmonary and non-laryngeal	2943 (54%)	2312 (53%)	N/a	N/a
Social risk factor				
History of homelessness	230 (4%)	130 (3%)	187 (7%)	90 (5%)
History of imprisonment	127 (2%)	76 (2%)	105 (4%)	50 (3%)
History of drug use	229 (4%)	141 (3%)	179 (7%)	101 (6%)
BCG vaccinated				
Yes	3136 (58%)	2627 (61%)	1541 (58%)	1115 (61%)
No	1117 (21%)	848 (19%)	568 (21%)	354 (19%)
Unknown	1129 (21%)	800 (19%)	563 (21%)	351 (19%)
Age				
15 years old or over	5367 (98%)	4046 (93%)	2641 (97%)	1658 (90%)
Under 15 years old	124 (2%)	284 (7%)	75 (3%)	187 (10%)
Home visits policy				
Yes	1179 (32%)	369 (9%)	907 (33%)	230 (13%)
No	3712 (68%)	3927 (91%)	1801 (67%)	1593 (87%)

### Indicator 1. Proportion of pulmonary index cases who had at least one contact identified

Of 2716 index cases with pulmonary disease, 2481 (91%) had at least one contact identified (table 2). This led to 12 248 contacts identified, a mean and median of 4.5 (95% CI 4.36 to 4.66) and 4 (IQR: 2–6) per index case, respectively. Table 3 shows the predictors of having at least one contact identified; the multivariable analysis is restricted to those with complete data on the variables included, limiting the sample from 2716 to 2327 of which 2168 (93%) cases had at least one contact identified. Male cases were less likely than female cases to have at least one contact identified (adjusted OR (aOR): 0.46 (0.30 to 0.68,)), as were those with a history of imprisonment compared with those without a history of imprisonment (aOR: 0.27 (0.14 to 0.52)), those of black African ethnicity compared with those of Indian ethnicity (aOR: 0.47 (0.27 to 0.83)) and recent migrants compared with long-term migrants (aOR: 0.54 (0.35 to 0.84)). Smear-positive index cases were more likely to have at least one contact identified compared with smear-negative index cases (aOR: 1.84 (1.20 to 2.82)). There was a significant positive linear association between the number of social care or directly observed therapy (DOT) workers (aOR per staff per 100 cases: 1.36 (1.07 to 1.72)) at the clinic and whether a case had at least one contact identified. These associations were similar for the proportion of index cases with three or more contacts named, or with five or more, except that index cases with a history of drug use were more likely to have three or more contacts named than those without (data not shown).

**Table 2:** Levels of indicators and outcomes over study period

Indicator	Number positive/total	Percentage (95% CI)
1. The proportion of pulmonary index cases who have at least one contact identified	2481/2716	91% (90% to 92%)
The proportion of pulmonary index cases who have at least three contacts identified	1810/2716	67% (65% to 68%)
The proportion of pulmonary index cases who have at least five contacts identified	1093/2716	40% (38% to 42%)
2. The proportion of identified contacts of pulmonary index cases who are evaluated	10 251/11 981	86% (85% to 86%)
3. The proportion of evaluated contacts that have active TB	294/16 495	1.8% (1.6% to 2.0%)
The proportion of evaluated contacts of pulmonary smear-positive index cases that have active TB	177/4328	4.1% (3.5% to 4.7%)
The proportion of evaluated contacts of pulmonary smear-negative index cases that have active TB	45/3421	1.3% (0.98% to 1.8%)
The proportion of evaluated contacts of non-pulmonary and non-laryngeal index cases that have active TB	51/7264	0.70% (0.51% to 0.89%)
4. The proportion of evaluated child contacts that have LTBI	490/4850	10% (9.3% to 11%)
The proportion of evaluated child contacts of pulmonary smear-positive index cases that have LTBI	277/1046	26% (24% to 29%)
The proportion of evaluated child contacts of pulmonary smear-negative index cases that have LTBI	93/980	9.5% (7.8% to 11%)
The proportion of evaluated child contacts of non-pulmonary and non-laryngeal index cases that have LTBI	78/2386	3.3% (2.6% to 4.0%)

LTBI, latent TB infection.

**Table 3:** Associations with pulmonary index cases having at least one contact named (indicator 1)

Characteristics of the index case	At least one contact identified			
	Yes (row %)*	No*	aOR (95% CI)	p Value
Total	2168 (93%)	159	N/a	N/a
UK born				
Yes	506 (94%)	31	1.16 (0.71 to 1.91)	0.01
No, long-term migrant	1390 (94%)	94	1	
No, recent migrant	272 (89%)	34	0.54 (0.35 to 0.84)	
History of drug use				
Yes	133 (92%)	11	1.57 (0.73 to 1.37)	0.25
No	2035 (93%)	148	1	
History of homelessness				
Yes	123 (85%)	21	0.56 (0.31 to 1.01)	0.06
No	2045 (94%)	138	1	
Former prisoner				
Yes	76 (81%)	18	0.27 (0.14 to 0.52)	<0.01
No	2092 (94%)	141	1	
Sex				
Male	1320 (91%)	124	0.46 (0.30 to 0.68)	<0.01
Female	848 (96%)	35	1	
Ethnicity				
Indian	417 (95%)	20	1	<0.01
Black-African	468 (89%)	59	0.47 (0.27 to 0.83)	
White	431 (93%)	34	0.73 (0.38 to 1.41)	
Other	852 (95%)	46	1.06 (0.61 to 1.85)	
Culture				
Positive	1684 (94%)	104	1	0.02
Negative	419 (90%)	46	0.59 (0.40 to 0.89)	
Not done	65 (88%)	9	0.52 (0.23 to 1.16)	
Smear				
Negative	945 (92%)	79	1	<0.01
Not done	367 (90%)	40	0.69 (0.44 to 1.06)	
Positive	856 (96%)	40	1.84 (1.20 to 2.82)	
Clinic case count (linear, 100 cases/year)	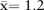	s=0.58	0.72 (0.46 to 1.12)	0.15
Clinic policy of home visits*				
Yes	714 (92%)	64	0.66 (0.44 to 0.99)	0.04
No	1454 (94%)	95	1	
Age of index case				
0–14 years	63 (97%)	2	1	0.19
15 years and over	2105 (93%)	157	0.36 (0.08 to 1.62)	
Notification rate*† (linear, 10 cases/100 000 population/year)	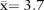	s=0.58	1.10 (0.98 to 1.23)	0.10
Nurses* (linear, nurses/100 cases/year)	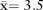	s=0.87	0.90 (0.63 to 1.28)	0.54
Admin staff* (linear, staff/100 cases/year)	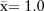	s=0.63	0.81 (0.50 to 1.31)	0.39
Social workers/DOT staff* (linear, staff/100 cases/year)	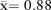	s=0.87	1.36 (1.07 to 1.72)	0.01

Adjusted OR (aOR) has been adjusted for everything in the table.

*Mean and SD presented for continuous variables.

†Notification rate in the year and local authority in which the index case was notified. N=2327.

DOT, directly observed therapy.

### Indicator 2. Proportion of contacts of pulmonary index cases that were evaluated

Of the 12 248 contacts identified, 11 981 had data on age and screening location and were included in this analysis. Of these, 10 251 (86%) were evaluated (table 2), with a mean and median of 3.9 (95% CI 3.72 to 4.00) and 3 (IQR: 1–5) per index case, respectively. The multivariable analysis of predictors of a contact being evaluated in table 4 is restricted to those with complete data on the variables included, limiting the sample of contacts from 11 981 to 10 476 of which 8986 (86%) were evaluated. Identified contacts of male index cases were less likely to be evaluated than those of female index cases (aOR: 0.68 (0.54 to 0.85)), as were contacts aged over 14 years (aOR: 0.30 (0.24 to 0.39)) when compared with those aged under 15 years. Contacts screened at the clinic of their index case (aOR: 1.65 (1.26 to 2.16)) were more likely to be evaluated than those screened elsewhere. Identified contacts of index cases notified in boroughs with higher notification rates (aOR per 10 cases per 100 000: 0.93 (0.87 to 0.99)), and contacts of index cases of white ethnicity (compared with Indian index cases, aOR: 0.61 (0.42 to 0.90)) were less likely to be evaluated, though these two associations were weaker.

**Table 4:** Associations with contacts of pulmonary index cases being evaluated (indicator 2)

Characteristics of the index case	Contact evaluated			
	Yes (row %)*	No*	aOR (95%CI)	p Value
Total	8986 (86%)	1490	N/a	N/a
UK born				
Yes	2240 (86%)	907	1.22 (0.92 to 1.61)	0.13
No, long-term migrant	5692 (86%)	357	1	
No, recent migrant	1054 (82%)	226	0.82 (0.59 to 1.13)	
History of drug use				
Yes	564 (80%)	137	0.99 (0.60 to 1.62)	0.97
No	8422 (86%)	1353	1	
History of homelessness				
Yes	480 (77%)	147	0.66 (0.40 to 1.06)	0.09
No	8506 (86%)	1343	1	
Former prisoner				
Yes	8708 (86%)	1400	0.63 (0.35 to 1.14)	0.13
No	278 (76%)	90	1	
Sex				
Male	5106 (83%)	1024	0.68 (0.54 to 0.85)	<0.01
Female	3880 (89%)	466	1	
Ethnicity				
Indian	1690 (87%)	263	1	0.05
Black African	1985 (88%)	275	0.88 (0.62 to 1.25)	
White	1739 (82%)	378	0.61 (0.42 to 0.90)	
Other	3572 (86%)	574	0.93 (0.69 to 1.25)	
Culture				
Positive	7221 (86%)	1174	1	0.46
Negative	1507 (84%)	280	0.84 (0.63 to 1.11)	
Not done	258 (88%)	36	1.03 (0.48 to 2.21)	
Smear				
Negative	3391 (84%)	625	1	0.14
Not done	1285 (85%)	220	1.02 (0.74 to 1.41)	
Positive	4310 (87%)	645	1.27 (1.00 to 1.61)	
Clinic case count* (linear, 100 cases/year)	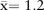	s=0.58	0.81 (0.64 to 1.04)	
Clinic policy of home visits				
Yes	3078 (87%)	480	1.09 (0.82 to 1.43)	0.56
No	5908 (85%)	1010	1	
Age of index case				
0–14 years	292 (90%)	34	1	0.93
15 years and over	8694 (86%)	1456	0.97 (0.53 to 1.80)	
Notification rate*† (linear, 10 cases/100 000 population/year)	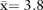	s=2.1	0.93 (0.87 to 0.99)	0.02
Nurses* (linear, nurses/100 cases/year)	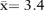	s=0.87	0.89 (0.72 to 1.09)	0.26
Admin staff* (linear, staff/100 cases/year)	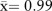	s=0.60	1.17 (0.86 to 1.60)	0.32
Social workers/DOT staff* (linear, staff/100 cases/year)	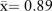	s=0.89	0.95 (0.81 to 1.10)	0.47
Age of contact				
15 years and over	6569 (83%)	1344	0.30 (0.24 to 0.39)	<0.01
0–14 years	2417 (94%)	146	1	
Contact screened at clinic				
Yes	7629 (87%)	1147	1.65 (1.26 to 2.16)	<0.01
No	1357 (80%)	343	1	

*Mean and SD presented for continuous variables.

†Notification rate in the year and local authority in which the index case was notified. N=10 476.

aOR, adjusted OR; DOT, directly observed therapy.

### Indicator 3. Proportion of evaluated contacts diagnosed with active TB

Of the 16 495 contacts (of index cases with disease at any site) evaluated, and no longer under investigation for signs of TB at the time of the study, 294 (1.8%, 95% CI 1.6% to 2.0%) were diagnosed with active TB (table 2). This figure rises to 2.6% (243/9213) (95% CI 2.3% to 3.0%) and 4.1% (177/4328) (95% CI 3.5% to 4.7%) for the contacts of pulmonary index cases and sputum smear-positive pulmonary index cases, respectively. This figure drops to 1.3% (45/3421) (95% CI 0.98% to 1.8%) and 0.70% (51/7264) (95% CI 0.51% to 0.89%) for the contacts of smear-negative pulmonary cases index cases and index cases without pulmonary or laryngeal involvement, respectively. Considering just index cases within the black African ethnic group (the ethnic group with the highest yield of active disease per contact) 2.8% (107/3817) (95% CI 2.3% to 3.3%) of evaluated contacts had active disease. For index cases aged under 15 years, 5.5% (27/491) (95% CI 3.5% to 7.5%) of their evaluated contacts had active disease. The multivariable analysis of predictors of an evaluated contact being diagnosed with TB in table 5 is restricted to those with complete data on the variables included, limiting the sample from 16 495 to 14 614 contacts of which 263 (1.8%) were diagnosed with TB. Adult contacts were associated with lower yields of active TB per contact (aOR: 0.55 (0.40 to 0.75)) (compared with child contacts). Additionally, index cases aged below 15 years, of black African ethnicity or with pulmonary or laryngeal disease (especially those who are smear-positive) were associated with contacts having an increased risk of active disease. None of the assessed social risk factors (history of homelessness, drug use or imprisonment) for the index case were associated with the contact having active TB.

**Table 5:** Associations with evaluated contacts (of index cases with TB at any site) with active TB

Characteristics of the index case	Contact diagnosed with active TB			
	Yes (row %)*	No*	aOR (95%CI)	p Value
Total	263 (1.8%)	14 351	N/a	N/a
UK born				
Yes	71 (2.3%)	2973	1.31 (0.86 to 1.99)	0.41
No, long-term migrant	159 (1.6%)	9834	1	
No, recent migrant	33 (2.1%)	1544	1.23 (0.71 to 2.15)	
History of drug use				
Yes	18 (2.9%)	612	1.06 (0.56 to 2.01)	0.85
No	245 (1.8%)	13 739	1	
History of homelessness				
Yes	13 (2.6%)	485	0.77 (0.36 to 1.66)	0.51
No	250 (1.8%)	13 866	1	
Former prisoner				
Yes	9 (2.8%)	312	1.02 (0.37 to 2.84)	0.96
No	254 (1.8%)	14 039	1	
Sex				
Male	164 (2.0%)	7884	1.35 (0.95 to 1.91)	0.09
Female	99 (1.5%)	6467	1	
Ethnicity				
Indian	39 (1.2%)	3130	1	<0.01
Black African	103 (3.0%)	3324	1.95 (1.17 to 3.26)	
White	32 (1.7%)	1910	0.81 (0.44 to 1.50)	
Other	89 (1.5%)	5987	1.03 (0.63 to 1.67)	
Culture				
Positive	225 (2.4%)	9316	1	0.12
Negative	71 (0.8%)	4033	0.70 (0.43 to 1.15)	
Not done	7 (0.7%)	1002	0.50 (0.23 to 1.09)	
Disease type				
Non-pulmonary and non-laryngeal	41 (0.6%)	6391	1	<0.01
Pulmonary smear-negative	44 (1.4%)	3107	2.10 (1.20 to 3.69)	
Pulmonary smear-positive	166 (4.3%)	3696	6.71 (4.11 to 11.0)	
Pulmonary, smear unknown or laryngeal	12 (1.0%)	1157	1.39 (0.73 to 2.64)	
BCG				
Not vaccinated	51 (1.6%)	3222	0.78 (0.51 to 1.18)	0.28
Vaccinated	177 (2.0%)	8502	1	
Unknown	35 (1.3%)	2627	0.73 (0.44 to 1.19)	
Clinic case count* (linear, 100 cases/year)	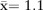	s=0.52	0.97 (0.68 to 1.40)	0.89
Age of index case				
0–14 years	25 (5.7%)	417	1	<0.01
15 years and over	238 (1.7%)	13 934	0.25 (0.13 to 0.48)	
Notification rate*† (linear, 10 cases/100 000 population/year)	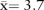	s=1.9	1.05 (0.95 to 1.17)	0.32
Age of contact				
15 years and over	151 (1.5%)	10 133	0.55 (0.40 to 0.75)	<0.01
0–14 years	112 (2.6%)	4218	1	
Contact screened at clinic				
Yes	233 (1.9%)	12 162	1.38 (0.81 to 2.37)	0.24
No	30 (1.4%)	2189	1	

*Mean and SD presented for continuous variables.

†Notification rate in the year and local authority in which the index case was notified. N=14 614.

aOR, adjusted OR.

### Indicator 4. Proportion of evaluated child contacts diagnosed with LTBI

Of the 4850 child contacts evaluated and no longer under investigation for signs of TB at the time of the study, 490 (10%, 95% CI 9.3% to 11%) were diagnosed with LTBI (table 2). This figure rises to 26% (277/1046) (95% CI 24% to 29%) for contacts of sputum smear-positive pulmonary index cases only, and drops to 9.5% (93/980) (95% CI 7.8% to 11%) and 3.3% (78/2386) (95% CI 2.6% to 4.0%) for the contacts of smear-negative pulmonary index cases and cases without pulmonary or laryngeal involvement, respectively. For index cases aged under 15 years, 17% (32/184) (95% CI 12% to 23%) of their evaluated child contacts had LTBI. The multivariable analysis of predictors of a child contact being diagnosed with TB in table 6 is restricted to those with complete data on the variables included, limiting the sample from 4850 to 4305 child contacts of which 440 (10%) were found to have LTBI. Pulmonary smear-negative (aOR: 2.92 (1.96 to 4.35)) and pulmonary smear-positive index cases (aOR: 8.39 (5.76 to 12.2)) (both relative to non-pulmonary and non-laryngeal index cases) were positively associated with child contacts having LTBI (table 6). Conversely, culture-negative index cases (aOR relative to culture-positive index cases: 0.51 (0.34 to 0.76)) were negatively associated with child contacts having LTBI. None of the assessed social risk factors (a history of homelessness, drug use or imprisonment) for the index case were associated with child contacts having LTBI, though the numbers were very small.

**Table 6:** Associations with evaluated child contacts (of index cases with TB at any site) being diagnosed with LTBI

Characteristic of the index case	Child contact diagnosed with LTBI			
	Yes (row %)*	No*	aOR (95% CI)	p Value
Total	440 (10%)	3865	N/a	N/a
UK born				
Yes	113 (16%)	611	1.23 (0.85 to 1.78)	0.44
No, long-term migrant	285 (9%)	2854	1	
No, recent migrant	42 (10%)	400	0.87 (0.51 to 1.48)	
History of drug use				
Yes	29 (18%)	136	1.35 (0.62 to 2.96)	0.45
No	411 (10%)	3729	1	
History of homelessness				
Yes	17 (14%)	108	0.95 (0.47 to 1.94)	0.90
No	423 (10%)	3757	1	
Former prisoner				
Yes	8 (10%)	74	0.53 (0.21 to 1.36)	0.19
No	432 (10%)	3791	1	
Sex				
Male	218 (10%)	1995	0.80 (0.59 to 1.09)	0.16
Female	222 (11%)	1870	1	
Ethnicity				
Indian	62 (8%)	700	1	0.41
Black African	134 (10%)	1232	1.07 (0.69 to 1.64)	
White	72 (18%)	322	1.54 (0.90 to 2.62)	
Other	172 (10%)	1611	1.06 (0.71. 1.58)	
Culture				
Positive	375 (14%)	2283	1	<0.01
Negative	49 (4%)	1260	0.51 (0.34 to 0.76)	
Not done	16 (5%)	322	0.60 (0.33 to 1.11)	
Disease type				
Non-pulmonary and non-laryngeal	67 (3%)	2041	1	<0.01
Pulmonary smear-negative	91 (10%)	823	2.92 (1.96 to 4.35)	
Pulmonary smear-positive	252 (27%)	692	8.39 (5.76 to 12.2)	
Pulmonary, smear unknown or laryngeal	30 (9%)	309	2.35 (1.35 to 4.10)	
BCG				
Not vaccinated	87 (10%)	822	0.83 (0.56 to 1.21)	0.54
Vaccinated	280 (11%)	2343	1	
Unknown	73 (9%)	700	1.08 (0.72 to 1.60)	
Clinic case count* (linear, 100 cases/year)	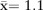	s=0.52	1.22 (0.85 to 1.76)	0.28
Age of index case				
0–14 years	30 (18%)	134	1	0.01
15 years and over	410 (10%)	3731	0.40 (0.20 to 0.77)	
Notification rate*† (linear, 10 cases/100 000 population/year)	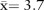	s=1.8	1.04 (0.93 to 1.16)	0.45
Contact screened at clinic				
Yes	388 (11%)	3306	1.29 (0.83 to 2.01)	0.27
No	52 (8.5%)	559	1	

*Mean and SD presented for continuous variables.

†Notification rate in the year and local authority in which the index case was notified. N=4305.

aOR, adjusted OR; LTBI, latent TB infection.

## Discussion

Our analyses provide the first estimates of indicators that will be used to monitor TB contact tracing in England, as part of the Collaborative Tuberculosis Strategy for England. We found that 91% of pulmonary TB cases in London had at least one contact identified and 86% of those contacts were investigated. For all index cases, 1.8% of evaluated contacts had active TB disease and 10% of child contacts had LTBI. The proportion of contacts diagnosed with TB or LTBI was almost fivefold greater for contacts of pulmonary or laryngeal index cases than for index cases with other disease types. Compared with female index cases, male index cases had fewer contacts identified, and fewer of those identified were evaluated, but sex had no significant effect on whether a case's evaluated contacts had LTBI or TB disease. Perhaps surprisingly, social risk factors were generally not significantly associated with either identifying contacts, evaluating those contacts, or for the resultant TB or LTBI yield per contact, the exception being that those with a prison history were less likely to have contacts identified compared with those without a prison history. The study may however have been underpowered to discern these relationships, as only around 5% of included index cases had each social risk factor. Contacts of children were more likely than those of adults to be diagnosed with TB; in the former circumstance, it is more likely that one of the contacts diagnosed with TB was the source case for that child.

By using data elicited through cohort review, we could include more information than that available from routine surveillance. One limitation was that data from several clinics for some periods had to be excluded as cohort review was done selectively (see online supplementary appendix). We mitigated this potential source of bias by only using data from clinics that reported 80% or more of their contacts, although some differences between included and excluded cases remained (table 1) and it reduced the power of the study. Also, cases removed from the multivariable analysis due to missing variables were more likely to be culture-positive, white, UK-born or homeless, and less likely to be screened at the clinic, than those included. It is difficult to predict the impact of these exclusions on the results. However, the proportion of contacts evaluated (86%) may be an overestimate, given that both more contacts of white index cases and fewer contacts screened at the clinic are excluded than included. Further, as more contacts of children were excluded than included (table 1), it is possible that the estimates of yield of TB and LTBI among contacts are underestimates. A second limitation is that little data were collected on individual contacts, potentially masking important determinants of contact outcomes, including the length and intensity of exposure, and risk factors increasing the chance of the contact progressing to disease. A third limitation is that the available data come from a relatively short period (42 months), so temporal trends were indiscernible. Unfortunately, data on the HIV status of index cases are not recorded in the LTBR. Finally, the number of patients with multidrug-resistant and extensively drug-resistant TB was too small to do a subgroup analysis of this group.

The proportion of pulmonary index cases with at least one contact identified (91%) compares favourably with previous figures for one sector of London in 2012, namely 78% and 88% before and after cohort review, respectively.^11^ It is also comparable to recent findings from the USA where the corresponding figures were 94% and 86% for smear-positive and smear-negative, culture-positive index cases, respectively,^24^ and higher than the figure in Piedmont, Italy (77%).^17^ Similarly, the proportion of identified contacts who were evaluated (88%) was higher than the north central London (74% precohort review, 82% postcohort review) and US figures (82% for smear-positives, 81% for smear-negatives). These figures suggest London TB clinics undertake high quality contact tracing, although further improvements in certain groups may be feasible. Note that the higher proportion of smear-positive index cases with at least one contact identified than smear-negative index cases, found in this study and others, is likely due to clinics' need to focus limited resources on those index cases for whom the yield is likely to be highest. The yield per contact of all forms of TB in this study was higher than from a similar study in Birmingham, another high incidence inner city region of the UK, in 1990–2010.^14^ That study found a yield per contact of active TB of 3.3% (compared with 4.1% in this study) and 0.58% (versus 0.70% in this study) for smear-positive pulmonary and non-pulmonary index cases, respectively. The yield per contact of active TB among contacts of pulmonary index cases presented here (2.6%) is high relative to other studies in high-income settings. A systematic review of contact tracing outcomes found a grouped yield per contact of TB in high-income countries of 1.4%,^8^ a similar study to ours in Italy found a yield per contact of active disease of 0.71%,^17^ and a study in Amsterdam found 0.79% of contacts had prevalent TB and 0.39% incident TB,^18^ although these comparisons do not account for differences in smear-positivity prevalence. There are no comparable estimates of the yield per contact of LTBI among child contacts. The overall prevalence among contacts (1.8%) compares to 0.50% of contacts with prevalent TB and 0.53% with incident TB in a European study.^19^

Overall, TB contact tracing in London is performing well across the suite of indicators when compared with previous studies in London or elsewhere. Contacts of cases with pulmonary or laryngeal TB are more likely to have TB or LTBI, compared with contacts of cases with non-pulmonary and non-laryngeal TB. However, prevalence of active TB among contacts of non-pulmonary patients is still very high (0.70%) relative to background TB prevalence, (0.027% in 2006^25^), which has implications for the recent change to the National Institute for Health and Care Excellence (NICE) TB guidelines, limiting screening to contacts of pulmonary and laryngeal patients.^7^

We found that contacts screened at a different clinic to the index case (accounting for 16% of all contacts) are less likely to be evaluated than those screened at the same clinic, suggesting gains can be made by improving cross-clinic contact tracing. Clinics were increasingly likely to successfully evaluate their contacts with increasing numbers of DOT and social workers per index case, indicating the important role played by these staff in building relationships with patients. Future continuation of these relationships may be affected by recent policy recommendations, for example, cessation of screening of contacts of non-pulmonary non-laryngeal patients and screening for LTBI in all contacts aged under 65 years (previously just under 35 years old).

Further work elucidating how performance against contact tracing indicators affects transmission and diagnostic delay would be important, as would the impact of recent changes in procedure to comply with latest national guidance. The recently changed NICE guidelines recommend limiting screening to pulmonary and laryngeal cases' contacts (bringing the UK into line with many other countries), as well as testing all those aged under 65 years with TST/IGRA and administering preventive therapy to this group (rather than just those aged under 35 years), but the implications for cost-effectiveness are unclear. Cost-effectiveness modelling using data from this study may help. Completion of cohort review fields by all clinics has improved recently; continued high levels of completion of these fields would greatly benefit future contact tracing studies. Further work to understand the full impact of home visits at an individual level would be useful. This intervention has been found to aid identification and evaluation of contacts in the USA^26^ and Portugal,^27^ and to improve preventive therapy outcomes in the USA and Canada,^28^ but our analysis was unable to show a relationship between home visits and improved contact identification and evaluation. Finally, qualitative research into improving engagement with men and those with prison history could potentially improve the proportion of contacts successfully evaluated, as these groups are less likely (compared with women and those without a prison history, respectively) to have contacts identified and evaluated (for men) but do not necessarily have lower yields per contact, suggesting room for improvement.
