# Supplementary material for: An evaluation of tuberculosis contact investigations against national standards
Source: Thorax. 2017 Apr 7;72(8):736–45. doi: 10.1136/thoraxjnl-2016-209677 (PMC5537523; doi:10.1136/thoraxjnl-2016-209677)
Supplement: Supplementary data [file thoraxjnl-2016-209677supp001.pdf]

Appendix

Table A: The percentage of all cases (i.e. those involving indicators 3 and 4) that were included in cohort review by sector and quarter. In this context, a case was assumed to have been presented at cohort review if at least one of the additional variables included as part of cohort review was completed. Shaded sections are those which were included in analyses, i.e. were more than 80% complete.

	3rd 2012	4th 2012	1st 2013	2nd 2013	3rd 2013	4th 2013	1st 2014	2nd 2014	3rd 2014	4th 2014	1st 2015	2nd 2015	3rd 2015	4th 2015
North Central	12%	97%	99%	99%	99%	98%	99%	99%	98%	99%	99%	100%	74%	71%
North East	8%	61%	12%	27%	24%	75%	89%	98%	100%	100%	100%	100%	67%	37%
North West inner	53%	96%	95%	96%	98%	93%	100%	96%	100%	100%	100%	99%	100%	79%
North West outer	29%	32%	29%	20%	42%	9%	80%	55%	62%	99%	93%	83%	89%	20%
South East	99%	100%	100%	100%	96%	98%	100%	98%	99%	99%	98%	99%	100%	49%
South West	0%	82%	100%	99%	100%	100%	100%	100%	85%	75%	95%	99%	100%	88%

Table B: The percentage of all pulmonary cases (i.e. for indicators 1 and 2) that were included in cohort review by sector and quarter. In this context, a case was assumed to have been presented at cohort review if at least one of the additional variables included as part of cohort review was completed. Shaded sections are those which were included in analyses, i.e. had a completeness of over 80%.

	3rd 2012	4th 2012	1st 2013	2nd 2013	3rd 2013	4th 2013	1st 2014	2nd 2014	3rd 2014	4th 2014	1st 2015	2nd 2015	3rd 2015	4th 2015
North Central	9%	95%	100%	98%	100%	96%	100%	98%	98%	97%	100%	100%	73%	77%
North East	14%	94%	22%	42%	30%	86%	97%	99%	100%	100%	100%	100%	66%	40%
North West inner	46%	96%	98%	93%	97%	91%	100%	97%	100%	100%	100%	100%	100%	73%
North West outer	50%	43%	43%	34%	76%	9%	91%	74%	84%	98%	91%	86%	91%	16%
South East	98%	100%	100%	100%	98%	98%	100%	96%	100%	98%	97%	100%	100%	42%
South West	0%	75%	100%	100%	100%	100%	100%	100%	82%	84%	96%	100%	100%	86%

Table C: The exposures included as potential determinants of each indicator.

Exposure	Identified	Evaluated	TB	LTBI
**Index born in UK**	Yes	Yes	Yes	Yes
**Site of disease & smear status of index**	Yes	Yes	Yes	Yes
**Index has history of homelessness**	Yes	Yes	Yes	Yes
**Index has history of drug use**	Yes	Yes	Yes	Yes
**Index has prison history**	Yes	Yes	Yes	Yes
**Index BCG vaccinated**	No	No	Yes	Yes
**Sex of index**	Yes	Yes	Yes	Yes
**Ethnicity of index**	Yes	Yes	Yes	Yes
**Culture status of index**	Yes	Yes	Yes	Yes
**Notification rate of local authority**	Yes	Yes	Yes	Yes
**Age of index**	Yes	Yes	Yes	Yes
**Home visit to index case**	Yes	Yes	No	No
**Contact evaluated at clinic**	N/a	Yes	Yes	Yes
**Contact an adult or child**	N/a	Yes	Yes	No*
**Nurses per case at notifying clinic**	Yes	Yes	No	No
**Admin per case at notifying clinic**	Yes	Yes	No	No
**DOT per case at notifying clinic**	Yes	Yes	No	No
**Number of cases per year at clinic**	Yes	Yes	Yes	Yes
**Total**	**15**	**17**	**14**	**13**

*Only include children, as this is the only group we know were tested for LTBI.
